# Radiomics in Lung Diseases Imaging: State-of-the-Art for Clinicians

**DOI:** 10.3390/jpm11070602

**Published:** 2021-06-25

**Authors:** Anne-Noëlle Frix, François Cousin, Turkey Refaee, Fabio Bottari, Akshayaa Vaidyanathan, Colin Desir, Wim Vos, Sean Walsh, Mariaelena Occhipinti, Pierre Lovinfosse, Ralph T. H. Leijenaar, Roland Hustinx, Paul Meunier, Renaud Louis, Philippe Lambin, Julien Guiot

**Affiliations:** 1 Department of Respiratory Medicine, University Hospital of Liège, 4000 Liège, Belgium; an.frix@chuliege.be (A.-N.F.); r.louis@chuliege.be (R.L.); 2 Department of Nuclear Medicine and Oncological Imaging, University Hospital of Liège, 4000 Liège, Belgium; fcousin@chuliege.be (F.C.); pierre.lovinfosse@chuliege.be (P.L.); rhustinx@chu.ulg.ac.be (R.H.); 3 The D-Lab, Department of Precision Medicine, GROW-School for Oncology, Maastricht University, 6229 Maastricht, The Netherlands; t.refaee@maastrichtuniversity.nl (T.R.); akshayaa.vaidyanathan@radiomics.bio (A.V.); philippe.lambin@maastrichtuniversity.nl (P.L.); 4 Department of Diagnostic Radiology, Faculty of Applied Sciences, Jazan University, Jazan 45142, Saudi Arabia; 5 Research and Development, Radiomics, 4000 Liège, Belgium; fabio.bottari@radiomics.bio (F.B.); wim.vos@radiomics.bio (W.V.); sean.walsh@radiomics.bio (S.W.); mariaelena.occhipinti@radiomics.bio (M.O.); ralph.leijenaar@radiomics.bio (R.T.H.L.); 6 Department of Radiology, University Hospital of Liège, 4000 Liège, Belgium; Colin.Desir@chuliege.be (C.D.); Paul.meunier@chuliege.be (P.M.)

**Keywords:** radiomics, artificial intelligence, lung diseases, precision medicine

## Abstract

Artificial intelligence (AI) has increasingly been serving the field of radiology over the last 50 years. As modern medicine is evolving towards precision medicine, offering personalized patient care and treatment, the requirement for robust imaging biomarkers has gradually increased. Radiomics, a specific method generating high-throughput extraction of a tremendous amount of quantitative imaging data using data-characterization algorithms, has shown great potential in individuating imaging biomarkers. Radiomic analysis can be implemented through the following two methods: hand-crafted radiomic features extraction or deep learning algorithm. Its application in lung diseases can be used in clinical decision support systems, regarding its ability to develop descriptive and predictive models in many respiratory pathologies. The aim of this article is to review the recent literature on the topic, and briefly summarize the interest of radiomics in chest Computed Tomography (CT) and its pertinence in the field of pulmonary diseases, from a clinician’s perspective.

## 1. Introduction

Described as the “high-throughput extraction of large amounts of image features from radiographic images” [1], radiomics is the subject of much research. This quantitative instrument is beginning to establish itself as a recognized imaging biomarker and paraclinical tool, serving both the fields of diagnosis and prognosis, along with predicting or monitoring response to treatment. The aim of radiomics is to extract quantitative, actionable information from standard-of-care medical images (computed tomography (CT), magnetic resonance imaging (MRI), positron emission tomography (PET), etc.), which are not easily visible or quantifiable with the naked eye, in order to build a model assessing clinical outcomes, including diagnostic, prognostic or predictive perspectives, to precisely identify and describe a pathological entity.

**Figure 1:**
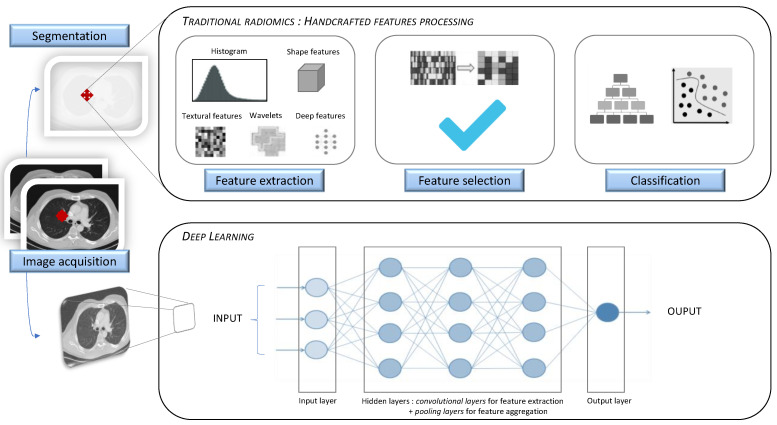
Traditional radiomics and deep learning approaches. Radiomic analysis workflow for handcrafted features (**top**) and deep learning method (**bottom**).

The following two methods can be used to perform a radiomic analysis: hand-crafted feature processing, or deep learning (DL). Hand-crafted feature (e.g., texture, shape, intensity, wavelet) processing will afford information on a specific targeted area of the imaging modality, distinctively from other related data (e.g., clinical, biological, genomic, histological, or treatment-related data). Contrastingly, deep learning methods will proceed to an extensive data-driven approach, processing a huge amount of information on the model of a simplified neural brain network, and without needing prior image segmentation (Figure 1).

The basic concepts inherent to radiomics and artificial intelligence are displayed in Table 1. Some of these concepts will be repeatedly used in the following sections.

As a young and developing tool in medicine, radiomics is still facing challenges that are limiting its wide use in clinical practice. Those include technical artifacts (inadequacy between acquisition and reconstruction, and inaccuracy in preprocessing procedures such as in segmentation), the lack of standard criteria to establish the accuracy of the results in the training and validation processes, and limited real-world experience in assessing the impact of quantitative imaging on clinical outcomes and diagnostic algorithm. Despite these limiting factors, research in the field is extensive, and should possibly and gradually establish radiomics as a clinical tool of major importance.

Applied to lung diseases, quantitative CT analysis extracts features such as threshold, histogram, and morphologic and texture analysis, and produces a quantifiable and reproducible evaluation of parenchymal changes. Radiomics has been used in the fields of nodules and cancer, obstructive and restrictive diseases, and infiltrative diseases (including idiopathic pulmonary fibrosis (IPF), hypersensitivity pneumonitis, connective tissue-related interstitial lung disease and combined pulmonary fibrosis and emphysema (CPFE)).

The aim of this narrative review is to report the recent literature, and briefly summarize the interest of radiomics and Artificial intelligence (AI) in chest CT and their application in the field of pulmonary diseases, from a clinician’s perspective.

## 2. The Role of Radiomics in Lung Diseases

The application of radiomics in the field of lung diseases could lead to improvements in the clinical and paraclinical workflow in diagnosis, prognosis, management, follow-up and monitoring the response to treatment.

### 2.1. Lung Nodules

Lung nodules appearance provides a substantial clinical challenge, raising the question of diagnosis, prognosis and management. The detection of small nodules is known to be a difficult task. Indeed, the current diagnostic classification relies on size and growth rate as the main differentiators between benign and malignant nodules [2,3,4]. However, this approach is still imperfect and needs to be improved. Furthermore, the final diagnosis still relies on invasive biopsy.

Different research groups among the quantitative imaging network (QIN) [5] are currently developing radiomic models to describe nodules or tumors, based on size, shape, margins, texture and intensity. Their aim is to assess if the lesion is malignant or benign, and to determine its inherent prognosis, to evaluate its response to treatment and eventually to correlate imaging with genomics or histology. The application of artificial intelligence and radiomics in pulmonary nodule management is promising [6]. Studies of interest concerning radiomics and lung nodules are described in Table 2.

*Diagnosis.* Apart from differentiating lung cancer from benign pulmonary nodules, thanks to nodule-size evaluation and texture-based analysis, radiomic analysis also extends its perspective to histological sub-typing among the same lesion, linking it to genomic information, and subsequently revealing prognostic and response to treatment evaluations. Several radiomic signatures that are able to accurately classify lung nodules have been published. For example, Chen et al. [7] defined a four-features radiomic signature, displaying an 84% accuracy in lung nodule classification. Additionally, the use of volumetric software leads to a more accurate and quantitative nodule sizing. In the same field, studying the volume doubling time (VTD) can lead to a better assessment of nodule growth rate. For example, a nodule with a VTD higher than 400–600 days has a 4.1% probability of malignancy, whereas a VTD lower than 400 days displays a malignancy probability of 9.7% [8]. Analysis of the NELSON trial results demonstrated that lung cancer mortality was significantly lower in high-risk patients who underwent volume CT screening (semi-automated extraction), than among those who underwent no screening [9]. Nodule texture can also be studied, empowering the accuracy with which radiomics can classify malignant and benign nodules. For example, Ma et al. [10] achieved an accuracy of 82.7%, and Hawkins et al. [11] demonstrated an accuracy of 80.0%. Other teams also reached relevant results [12,13]. Mao et al. [14] reported an overall accuracy of 89.8% in the qualitative diagnosis of small solitary pulmonary nodules (SSPNs), which outperformed the American College of Radiology (ACR) Lung-RADS approach [15]. Moreover, when texture analysis was combined to clinical and CT features, Lee et al. [16] demonstrated an improvement in the model performance (area under the curve, AUC), from 0.79 (clinical and CT features alone) to 0.93 (texture analysis incorporating clinical and CT features). Interestingly, there is a good concordance between the diagnostic abilities of radiomics software using ultra-low-dose chest CT compared to full-dose chest CT for lung nodule risk assessment, even if those are preliminary results [17]. Furthermore, Maldonado et al. [18] implemented and validated the BRODERS classifier (benign versus aggressive nodule evaluation using radiomic stratification), a high-resolution CT (HRCT)-based radiomic classifier in the characterization of indeterminate pulmonary nodules. Early works on convolutional neural networks (CNNs) compared to computer-aided detection/diagnosis (CAD) demonstrated a superiority of the radiomic approach in nodule classification, with a decrease in false positives, possibly reducing the need of several follow-ups [19,20,21]. Mehta et al. combined biomarkers, volumetric radiomics, and 3D CNNs to reach an algorithm classifying lung nodules [22]. Concerning histological subtyping, numerous studies have demonstrated the correlation between radiomic features and histology. For example, Wu et al. [23] described 53 radiomic features significantly associated with tumor histology, thus leading to an AUC of 0.72 in predicting the histological subtype. As discussed below (see “Section 2.2”), the possibility of radiomics to accurately predict the underlying gene expression of an identified tumor is also gathering considerable attention in recent years.

*Prognosis.* It has been shown that radiomic analysis performs well in the identification of nodules that are more at risk of evolution towards cancer. This has direct clinical implications, as it means shorter follow-up CT imaging and early detection of lung cancer. According to Digumarthy et al. [24], temporal changes in the radiomic features (process called delta-radiomics) of subsolid lung nodules indicates malignant etiology over benign. Indeed, they demonstrated that the radiomic features of benign nodules were stable over time, whereas the radiomic features of malignant nodules changed significantly between the baseline and follow-up CT, thus improving the accuracy up to 70.8% in the distinction between malignant and benign nodules with follow-up temporal changes.

One of the open questions that still remains is the sensitivity of quantitative imaging, and the correlation between imaging features computed with different segmentation algorithms. Indeed, segmentation algorithms ought to be highly reproducible, as the data extracted will serve for clinical purposes. Moreover, different segmentations might affect the radiomic features extraction. By performing a multicentric study based on a common set of reference images, Jayashree et al. [25] demonstrated a high correlation between groups of features (e.g., size and intensity features), whereas specific features within these groups did not correlate (e.g., reporting or not the size of airspaces within the lesion, affecting the mean intensity of the total nodule), uncovering subtle differences in the approach and calculations among the different centers. More research needs to be conducted to develop robust segmentation methods to provide accurate and reproducible nodule segmentation [26].

### 2.2. Cancer

Lung cancer is still the leading cause of death among neoplastic diseases in men and women worldwide [27]. The National Lung Cancer Screening Trial (NLST) demonstrated that CT screening in current and ex-smokers provides a significant survival benefit [28,29]. As of today, biopsies are still needed to establish the diagnosis and status of tumors. Nevertheless, this procedure is invasive and only reflects the characteristics of the part of the tumor from which the sample was obtained, considering that some tumors can be anatomically heterogeneous. Due to technological advances in AI, radiomic analysis could be seen as a virtual biopsy tool, and could have the potential to diagnose and determine tumor phenotypes. Radiomics has been used to assess tumor phenotypes using various imaging modalities, such as CT, MRI and PET CT [30]. Studies of interest, concerning radiomics and lung cancer, are described in Table 3.

*Diagnosis*. Several studies have demonstrated the potential of radiomics in lung cancer diagnosis and staging [23,31,32,33,34]. Beyond anatomical characterization, radiomics could be used to predict the presence of particular mutations in genes. For example, Liu et al. and Zhang et al. [35,36] established a correlation between CT radiomic features and EGFR (epidermal growth factor receptor) mutation, whereas Rios Velazquez et al. [37] created a radiomic model to classify mutations in pulmonary adenocarcinoma. Weiss et al. [38] assessed the potential of textural analysis to radiologically differentiate K-RAS mutations from pan-wildtype tumors, reaching an accuracy of 89.6%. In the same perspective, Tang et al. [39] defined 12 robust radiomic features, generating an immune-pathology informed model to predict immune modulator status (interesting Cluster of differentiation 3 (CD3) and Programmed death-ligand 1 (PDL1)). Lastly, hypothesizing the fact that radiomics could provide histopathological analysis, while having the advantage of being non-invasive, Wu et al. [23] performed radiomic analysis to predict the histopathological types of non-small cell lung carcinoma (NSCLC), reaching a correlation with tumor histology of 0.72 (AUC). Additionally, another study demonstrated that separating ground-glass and solid CT radiomic features of part-solid nodules was useful in diagnosing the invasiveness of lung adenocarcinoma. Their radiomic model based on ground-glass and solid features yielded an AUC of 0.98 on the test data set, which was significantly higher than five other models tested (Brock University model, clinical semantic model, volumetric models, radiomic signature based solely on gross tumor volume (GTV) features, and perinodular features) [40].

*Prognosis*. Numerous studies corroborated the correlation between radiomic features and prognosis, in terms of the survival or occurrence of distant metastases [26,31,41,42,43,44,45]. For instance, Mattonen et al. [46] demonstrated the accuracy of radiomics to predict local recurrence in patients with early stage NSCLC, treated with stereotactic ablative radiotherapy. Their results also suggested that radiomics could detect early changes in the tumor, associated with local recurrence, which would not have been taken into account by clinicians.

*Therapy*. The use of radiomics to predict response to therapy was explored by several research groups, but has not yet been translated to clinical use [47,48]. Coroller et al. [49] studied pre-treatment radiomics data to determine if they could have predicted the response after neoadjuvant therapy in patients with locally advanced NSCLC. They found seven radiomic features that were predictive of residual disease (AUC > 0.6), and one radiomic feature that was predictive of a complete response (AUC 0.63). Similarly, Kim et al. [50] used radiomic analysis in combination with conventional clinical features to predict the response to tyrosine kinase inhibitors (TKI) in epidermal growth factor receptor (EGFR) mutant NSCLC, achieving a good predictive performance, with a concordance index of 0.77.

Despite facing limitations inherent to its novelty (see “Section 3”), radiomics is seen as a revolutionary precision medicine approach in lung cancer. Its applications, as follow, in the field of research are broad and extensive: diagnosis, staging and prognosis, prediction of treatment response, and disease monitoring [51]. These characteristics are highly interesting, as lung cancer can face a rapid progression, but studies are still needed to reach real-life clinical application.

### 2.3. Obstructive Lung Diseases

In current clinical practice, pulmonary function tests are crucial to assess the characteristics of obstructive lung diseases. However, while being useful in assessing respiratory performance, as well as volume and resistance ranges, they cannot inform the clinician about the local extent of emphysema or air trapping. Overcoming this anatomical deficiency, quantitative CT analysis can be used, and extensive research has been carried out to automate the quantification of emphysema or air trapping severity and distribution [53,54], as well as to characterize airway diseases more precisely [55].

In this way, quantitative CT analysis and radiomics could be applied to various obstructive lung diseases, such as in the characterization of chronic obstructive pulmonary disease (COPD) or asthma, the detection of bronchiolitis obliterans, or even in planning eventual emphysema reduction therapy.

In obstructive pulmonary diseases, the lung texture and density are influenced by increased air abundance, compared to normal lungs. The origin of this excess of air plethora can be anatomical (emphysema) or functional (air trapping). In addition, the study of lung texture and density is highly biased by the respiratory phase [56]. During inspiration, the CT appearance of emphysema can be characterized by the following two methods: areas with a parenchyma density < −950 HU (emphysema index: percentage of parenchyma below attenuation threshold of −950 HU) [57], or areas related to the lowest 15th percentile (emphysema index: lung voxels below threshold value in HU, for which 15% of all lung voxels have a decreased attenuation value on the attenuation histogram) [58]. During expiration, air trapping can be defined as the area with a parenchyma density < −856 HU on expiratory CT. However, it might be difficult to differentiate low attenuation from air trapping versus low attenuation from emphysema. To address this issue, Pompe et al. [59] applied parametric response mapping (PRM), a method using a combination of threshold-based measures taken simultaneously during inspiratory and expiratory phases on co-registered CT, allowing a biphasic characterization of voxels.

Parallel to parenchyma characterization, quantitative analysis of airways can be realized up to the fifth bronchial generation. Quantitative CT metrics of airways include the study of wall thickness, area and density, and lumen diameter and area. However, its use faces a certain number of limitations, as airway metrics are highly influenced by lung volume, aging and inflammation [60]. Therefore, applying airway metrics in clinical practice is still at the preliminary stage.

#### 2.3.1. COPD

*Diagnosis*. Applying radiomics could lead to better COPD characterization and quantification [61,62]. Lynch et al. [63] used an integrative description of the visual and quantitative evaluation of CT images in COPD to determine COPD phenotypes, and classified them into emphysema-predominant subtypes (six different subclassifications) and airway-predominant subtypes (two subclassifications). Beyond the classical anatomical characterization, several research groups demonstrated the potential of CT radiomics features to correlate with lung function [52,64].

*Prognosis.* Cho et al. performed a radiomic analysis to predict survival and apply risk stratification in COPD, and reached a five-feature model displaying a C-index of 0.774, accurately identifying patients with an increased risk of mortality [65]. Interestingly, CT vascular features can also be helpful in the characterization of COPD, as the quantitative assessment of pulmonary vascular alterations in COPD patients exhibited correlations with clinical parameters, such as pulmonary function tests (PFTs) and survival, in the The Korean Obstructive Lung Disease (KOLD) cohort [66].

*Therapy*. Quantitative CT analysis can also be used to assess the progression of emphysema in alpha-1-antitrypsin deficiency and the response to augmentation therapy [67]. Moreover, the assessment of lung lesions in emphysema, by CT quantification and perfusion scintigraphy, implements the best prediction of outcome in lung volume reduction (LVR) as a therapeutic option [68,69].

#### 2.3.2. Asthma

Asthma phenotyping is of utmost importance for disease categorization and personalized treatment. In this context, airway remodeling is seen as a possible imaging biomarker. Quantitative imaging led to the definition of asthma clusters, which were found to respond differently to the bronchodilator between the different imaging clusters [70]. Further characterization has been possible with the quantification of air trapping. For example, Choi et al. [71] found that four radiological clusters had differences in their response to high-dose inhaled corticosteroids (ICS). Quantitative CT analysis in asthma can also be used as a novel marker to predict or assess the response to treatment, which can lead to more personalized therapy [71,72].

### 2.4. Interstitial Lung Diseases

As a heterogeneous group of pathologically distinct processes, but sometimes radiologically overlapping entities, interstitial lung diseases (ILD) can represent a diagnostic challenge and face an unpredictable clinical course. Combined to biological data and PFTs, thin-section chest CT is essential in differentiating interstitial lung diseases, evaluating their severity and evolution over time, and possibly monitoring their response to therapy. Nevertheless, visual assessments of thin-section CT and traditional PFTs evaluation are relatively insensitive to slight changes or early disease. Moreover, visual evaluation of HRCT pattern is highly subjective and variable, even among experts. The finest analysis of specific radiological patterns, such as ground-glass opacities, honeycombing, traction bronchiectasis, attenuation and reticular density, and their volumetric distribution and spatial relationships, can lead to more precise diagnosis. Therefore, the contribution of radiomics, as a reproducible and accurate imaging tool, is a major issue. Some studies also reported a strong correlation between radiomic features in ILD and pulmonary function tests. Studies of interest, concerning radiomics and interstitial lung diseases, are described in Table 4.

*Diagnosis.* Many studies have demonstrated the performance of quantitative CT analysis and its potential to assess the severity of ILD [73,74]. In IPF, Stefano et al. [75] demonstrated a strong correlation between radiological features and disease severity (*p* = 0.009). In scleroderma-related ILD, Martini et al. [76] used radiomics to detect ILD in sclerodermic patients and to predict their GAP (Gender, Age, Physiology) stage, as generally used in ILD evaluation (AUC 0.96). Many teams also determined correlations between quantitative radiological features and baseline PFTs [77,78]. One remaining recurrent matter of concern is the discernment between IPF and fibrosing non-specific interstitial pneumonia (NSIP). A recent study addressed this issue by using a CALIPER tool (computer-aided lung informatics for pathology evaluation and rating), combining PFTs and quantitative imaging to significantly discriminate NSIP from IPF [79]. Among other diagnostic issues, identifying the nature of mediastinal lymphadenopathy without recourse to a biopsy, in order to differentiate sarcoidosis and tuberculosis, remains challenging. Lee et al. [80] used quantitative imaging to discriminate sarcoidosis from tuberculosis lymphadenopathy, displaying significant differences in quantitative CT features between the two groups. All these studies make us consider the distant utopia of reaching a fully virtual biopsy, in which no tissue sample would be needed to obtain the same histological information, even if research and its clinical application are still at a preliminary stage.

*Prognosis*. The comparison of changes in radiological features led many teams to prove that radiomic features could predict the evolution of lung function, disease progression or mortality in ILD. In their study, by evaluating quantitative CT indexes and lung function, Best et al. [81] showed that forced vital capacity (FVC) and fibrosis index were predictors of short-term mortality in IPF, whereas more precise features (fibrosis index, mean lung attenuation, skewness and kurtosis) predicted disease progression. This is in line with the findings of Kim et al. [78], who demonstrated that quantitative lung fibrosis (QLF) score correlated well with changes in PFTs and disease progression in IPF. In the same perspective, other teams [82,83] used the CALIPER tool (computer-aided lung informatics for pathology evaluation and rating) to predict survival in IPF.

*Therapy*. Concerning the monitoring of response to therapy in ILD, few studies are currently available. Proving the usefulness of radiomics in this precise field, Kim et al. [84] assessed the efficacy of cyclophosphamide in scleroderma-related ILD, by using texture-based scores determining the QLF score. They established a significant change in QLF score after treatment, supporting the efficacy of cyclophosphamide over placebo, and also demonstrated a significant association between changes in QLF score, forced vital capacity (FVC) and dyspnea score. These few results, already promising, are the first signs of what radiomics could bring in the precise and quantifiable evaluation of the response to treatment in ILD.

### 2.5. Vascular Lung Diseases

The application of artificial intelligence in the field of vascular lung diseases is still at the preliminary stages. Only a few studies report results and mainly focus on pulmonary hypertension (PH). For example, Kiely et al. [85] managed to apply artificial intelligence in order to achieve a predictive model, using existing and real-world data to determine patients at high risk of idiopathic pulmonary hypertension, resulting in 99.99% specificity and 14.10% sensitivity.

However, it is strongly believed that AI and machine learning could be of high interest in the diagnostic and prognostic classification of PH. For instance, one area of research is the accurate segmentation of cardiac chambers on MRI or CT, and the segmentation of the pulmonary vascular network [86,87]. One notable current limitation of imaging is the inability to properly assess distal pulmonary arterial vasculature, which is the pathological site interesting pulmonary arterial hypertension (PAH). Therefore, applying radiomics on CT or MRI imaging could lead to a more accurate evaluation of pulmonary perfusion [88]. Lastly, as PH diagnosis still relies on right heart catheterization (RHC), any non-invasive diagnostic tool could be highly welcomed. Lungu et al. [89] hypothesized that combining mathematical and cardiopulmonary metrics with AI classifiers could add diagnostic value. Their classifier showed that 92% of patients were correctly classified, which led to the conclusion that combining the PH biomarker with AI classification algorithms enhanced the diagnostic performance of non-invasive techniques in PH.

### 2.6. Pleural Diseases

The rare studies exploring the pleura from a radiomic perspective only concern pleural tumor invasion in lung cancer. For example, Yang et al. [90] exposed a strong association between tumor imaging phenotype, as defined by radiomic features, and dry pleural dissemination. Studies are still needed in the field of radiomics, applied to benign or malignant primitive pleural diseases.

## 3. Challenges and Limitations

Being a novel and developing tool in medicine, radiomics is still inevitably facing challenges, limiting its wide use in clinical practice. These pitfalls can be found in most of the following steps of the radiomic workflow: from image acquisition to image segmentation, feature extraction, statistical analysis, and implementation in algorithms and real-life experience [51,91].

- Image acquisition: There is a disparity in acquisition parameters, as there are no real standardized imaging protocols. For example, there are differences in dose administration, reconstruction kernels, section thickness between the different imaging centers, and modalities. Moreover, variations in inspiratory effort can modify lung attenuation and volume, possibly leading to misinterpretation, affecting both threshold- and histogram-based quantification.
- Image segmentation: There is an inevitable intra- and inter-observer variability in manual segmentation methods, which could be improved by the use of semi- or fully automatic techniques. Similarly, intra-lesional heterogeneity can be a real challenge for accurate segmentation, as well as motion artifacts or noisy background due to low-dose scanning.
- Feature extraction: There is a risk of confusion between the signal of the ROI and the background noise, which could be improved by the development of filtering techniques, or by resampling strategies. Moreover, feature selection is facing disparities imputable to human error, which could be improved by the implementation of deep learning methods such as CNN.
- Statistical analysis: There is a disproportion between the tremendous number of possible features and the small population, generating a high rate of false positives (elegantly called “curse of dimensionality” [51]), which could be improved by the development of statistical corrections or cross-validation.
- Implementation and reproducibility: There is a lack of reproducibility between research groups, which could be improved by increasing the access to full data, extraction software and statistical methods. Another example of a limitation is misinterpretation from a trained algorithm. Indeed, a specific algorithm can only define a disease for which it was trained, possibly leading to the false suggestion of diseases sharing some common features.
- Robustness and Explainability of Artificial Intelligence: There are numerous issues to be addressed, concerning the application of AI in real life. For example, the use of extensive data in the development of machine learning models does not imply the automatic understanding of underlying mechanisms linking data. Moreover, AI systems can face concerns regarding reliability, as they may accumulate edge cases that are not taken into account by the algorithm. Lastly, the question of data protection must be raised, as potential matters concerning confidentiality can surface [92].

Recognition of the current limitations of radiomics is essential to avoid misleading to inappropriate or non-reproducible models. For example, in this perspective, Ibrahim et al. [93] proposed a workflow for accurate radiomic analysis. Standardization of imaging acquisition, segmentation, feature extraction, and calculation is fundamental to ensure robustness and dissemination of radiomics as a paraclinical tool in medicine.

## 4. Conclusions and Perspective

At the era of precision medicine, where personalized work-up and treatment according to individual variability is unavoidable, imaging biomarkers integrating information from extensive data is seen as a revolution. Radiomics is cementing its position as a promising tool in lung diseases, integrating data from imaging, clinical, histological and genomic information. Significant effort is being put into the investigation and resolution of its inherent limitations. The research is extensive and aims to progressively lead to its clinical application and routine utilization in real-life practice.

## Figures and Tables

**Table 1:** AI and radiomics: basic concepts.

Terminology Used in Radiomics and AI	
Artificial intelligence	Wide-ranging branch of computer science, generating complex software that perform tasks that would typically have required human intelligence, by sensing and responding to a feature of their environment.
CAD (Computer Aided Detection or Diagnosis)	Technology combining elements of artificial intelligence with radiological and pathology image processing. Its aim is to assist in the detection and/or diagnosis of diseases, improving the accuracy of radiologists with a reduction in time in the interpretation of images.
Radiomics	Method that extracts a large number of quantitative features from radiographic medical images using data-characterization algorithms, to help in disease diagnosis and prognosis.
Machine Learning	Field in artificial intelligence studying computer algorithms that improve automatically through experience, by building a model based on sample data, known as “training data”, in order to make predictions or decisions. Supervised learning: The computer receives example inputs and their foreseen outputs. Its goal is to learn a general and reproducible function that links inputs to outputs. Unsupervised learning: The computer receives no labels to the learning algorithm for previously undetected patterns in a data set, leaving it on its own to find structure in its input.
Convolutional neural networks	Class of deep neural networks, which have the particularity of being fully connected networks. It gives them the advantage of understanding the hierarchical pattern in data and assembling more complex patterns using smaller and simpler patterns.
Voxel	Single sample, or data point, on a regularly spaced, three-dimensional grid. In CT scans, the values of voxels are Hounsfield units. A voxel is a 3D pixel.
ROI (Region of Interest)	Image areas containing the information relevant to image processing.
Skew of histogram	Measure of the asymmetry of attenuation distribution. The lung normal attenuation histogram is skewed to the left. There is a decreased leftward skewness in IPF.
Kurtosis of histogram	Measurement of how sharp an attenuation distribution curve is. Kurtosis is abnormally low in idiopathic pulmonary fibrosis (IPF).
Threshold measurement	Total count of pixels/voxels above or below a specific attenuation value that determines a relative volume. Threshold measures in emphysema quantifies the extent of emphysema according to a specific index of −950 Hounsfield units (HU).
Texture analysis	Statistical methods that evaluate spatial relationship between voxels in an ROI, in order to characterize textural features of the parenchyma and give information about heterogeneity.

AI: Artificial Intelligence, CT: Computed Tomography, ROI: Region of Interest.

**Table 2:** Radiomics and lung nodules.

Study	Description	Cohort	Performance
Chen et al. (2018) [7]	- 750 extracted features, among which 76 relevant features were selected - 4-feature signature - Aim: nodule characterization	33 benign CT 42 malignant CT	Benign vs. malignant Accuracy 84% Sensitivity 92.85% Specificity 72.73%
De Koning et al. (2020) [9]	- 15,792 patients, divided into a screening group (T0–T1 year–T2 years–T3 years) and a no-screening group - Follow-up of 10 years - Aim: nodule characterization through volume and VTD	15,792 patients	Benign vs. malignant: impact on mortality At 10 years, cancer mortality = 2.5 deaths/100,000 persons/years (screening group) vs. 3.3 deaths/100,000 (no-screening group) Cumulative ratio 0.76 (*p* = 0.01)
Ma et al. (2016) [10]	- 583 extracted features - Random forest classifier - Aim: nodule characterization	36 benign CT 94 malignant CT	Benign vs. malignant Accuracy 82.7% Sensitivity 80% Specificity 85.5%
Hawkins et al. (2016) [11]	- 219 extracted features, among which 23 showed concordance correlation > 0.95 - Aim: nodule characterization	328 benign CT 170 malignant CT	Benign vs. malignant Accuracy 80%
Huang et al. (2018) [12]	- 1108 extracted features - Aim: nodule characterization	Training cohort 70 benign CT 70 malignant CT Validation cohort 26 benign CT 20 malignant CT	Benign vs. malignant Accuracy 91% Sensitivity 95% Specificity 88%
Uthoff et al. (2020) [13]	- Extracted features from nodule and perinodular parenchyma tissue - Aim: nodule characterization	Training cohort 289 benign CT 74 malignant CT Validation cohort 50 benign CT 50 malignant CT	Benign vs. malignant Accuracy 98% Sensitivity 100% Specificity 96%
Mao et al. (2019) [14]	- 385 extracted features - Comparison of radiomic model versus model of ACR Lung-RADS - Aim: nodule characterization	Training cohort 156 benign CT 40 malignant CT Validation cohort 75 benign CT 23 malignant CT	Benign vs. malignant Accuracy 89.8% Sensitivity 81% Specificity 92.2%
Maldonado et al. (2021) [18]	- 8-feature signature - Aim: to validate the BRODERS classifier (benign versus aggressive nodule evaluation using radiomic stratification) as a HRCT-based classifier for indeterminate pulmonary nodules	Validation cohort 91 malignant CT 79 benign CT	Benign vs. malignant AUC 0.90 Sensitivity 92.3% Specificity 62%
Mehta et al. (2021) [22]	- 1018 nodule CTs, malignancy rating from 1 to 5 according to volume - Fully supervised and semi-supervised classifiers - Aim: to reach an hybrid algorithm to estimate nodule malignancy by combining imagery and biomarkers/volumetric radiomic features	1018 CTs Malignancy rating from 1 to 5	Benign vs. malignant AUC 0.87 on fully supervised 3D CNN + random forest model (images, biomarkers and volumetric features) AUC 0.93 on semi-supervised random forest (biomarkers only)
Digumarthy et al. (2019) [24]	- 92 extracted features - 2 significant features at baseline - 52 significant features at follow-up - Aim: nodule characterization according to temporal changes	31 benign CT 77 malignant CT	Benign vs. malignant according to temporal changes AUC 0.741
Lee et al. (2014) [16]	- Clinical, thin-section CT and texture features - Aim: prediction of transient vs. persistent pattern of nodule	Transient PSNs 39 benign CT Persistent PSNs 17 benign CT 30 malignant CT	Prediction of persistent part-solid nodules AUC 0.93 if texture analysis was combined to clinical and CT features
Autrusseau et al. (2021) [17]	- >1000 extracted features - Aim: to compare quantitative and qualitative concordance of pulmonary nodule risk assessment by radiomic software between full-dose (FD) chest CT and ultra-low-dose (ULD) chest CT	99 lung nodules - FD chest CT imaging - ULD chest CT imaging	Concordance between FD and ULD chest CT in radiomic-guided nodule risk assessment ICC of 0.82, displaying a good agreement in malignancy similarity index between ULD and FD chest CT

CT: Computed Tomography, VTD: Volume Doubling Time, ACR: American College of Radiology, BRODERS: Benign versus aggRessive nODule Evaluation using Radiomic Stratification, HRCT: High Resolution Computed Tomography, AUC: Area under the Curve, CNN: Convolutional Nerual Network, ICC: Intraclass correlation coefficient, ULD: Ultra Low Dose, PSNs: Part-solid Nodules, FD: Full Dose.

**Table 3:** Radiomics and lung cancer.

Study	Description	Cohort	Performance
Wu et al. (2016) [23]	- 440 extracted features - 53 features associated with tumor histology - Aim: to predict cancer histopathology	Training cohort 198 malignant CT Validation cohort 152 malignant CT	Tumor histology correlation AUC 0.72
Yu et al. (2019) [34]	- 9 relevant features selected - Aim: to diagnose and predict pathologic stage in NSCLC	Training cohort 87 NSCLC CT Validation cohort 58 NSCLC CT	Diagnosis and staging in NSCLC AUC > 0.70, with predictive accuracy higher in lung adenocarcinoma than in lung squamous cell carcinoma
Liu et al. (2016) [36]	- 219 extracted radiomic features, among which 59 robust features were selected - Aim: search for correlation with EGFR mutation status in adenocarcinomas	298 malignant CT	Prediction of mutation status AUC EGFR+ status prediction 0.647, improved to 0.709 when adding a clinical model
Rios Velasquez et al. (2017) [37]	- 26 relevant features selected - Aim: search for correlation with KRAS and EGFR mutation status in adenocarcinomas	Training cohort 353 malignant CT Validation cohort 352 malignant CT	Prediction of mutation status AUC EGFR + versus EGFR− status 0.70 AUC KRAS + versus KRAS− status 0.63 AUC EGFR+ versus KRAS+ status 0.80
Tang et al. (2018) [39]	- Pathology markers studied: CD3 count and %PDL1 - 490 extracted features, among which 12 robust features were selected, then targeted into 4 features to generate 4 clusters (immune-pathology informed model) - Aim: to predict immune modulator status in NSCLC	Training cohort 114 malignant CT Validation cohort 176 malignant CT	Prediction of immune modulator status Favorable outcome in low CT intensity and high heterogeneity with low PDL 1 and high CD3
Wu et al. (2020) [40]	- 18 relevant features selected - Comparison of radiomic models (ground-glass and solid features) with other models (Brock model, clinical semantic and volumetric models) - Aim: to predict invasiveness of lung adenocarcinoma by using ground-glass and solid features from part-solid nodules	Training cohort 229 NSCLC Validation cohort 68 NSCLC	Prediction of invasiveness AUC 0.98 for the model combining ground-glass and solid features Improvement of 0.14 in AUC when adding ground-glass radiomic features to solid features
Coroller et al. (2015) [41]	- 445 extracted features, among which 35 relevant features were selected - Aim: to determine the capability of radiomic analysis to predict distant metastasis	Training cohort 98 malignant CT Validation cohort 84 malignant CT	Prediction of distant metastasis A multivariate radiomic signature (3 features) yielded a high prognostic performance for distant metastasis (CI 0.61)
He et al. (2019) [43]	- 519 extracted features, among which 35 relevant features were selected - Aim: to predict lymph node metastasis in resectable NSCLC	Training cohort 423 NSCLC CT Validation cohort 294 NSCLC CT	Prediction of lymph node metastasis Good discrimination for the model defining a radiomics-based predictive score (C index 0.785)
Ferreira et al. (2018) [45]	- 2777 extracted features, among which 100 most relevant features were selected - Aim: to predict lung cancer histopathology and metastases using machine learning models	Training cohort 52 malignant CT Validation cohort 16 malignant CT	Histology and distant metastasis AUC lymph nodal metastasis 0.89 AUC distant metastasis 0.97 AUC histopathology 0.92
Mattonen et al. (2016) [46]	- 104 extracted features, among which the 5 most relevant features were selected - Aim: to assess physicians’ ability to detect local recurrence versus radiomic tool	182 malignant CT	Prediction of recurrence after SBRT AUC 0.85 (radiomic signature of 5 features predicting local recurrence)
Coroller et al. (2016) [49]	- 15 relevant radiomic features selected - Aim: to assess if radiomics can predict response after neoadjuvant chemoradiation (NCT) in locally advanced NSCLC	127 malignant CT Training cohort 80% Validation cohort 20%	Prediction of response after NCT AUC for pathologic gross residual disease prediction (7 features) > 0.6 AUC for pathologic complete response (1 feature) 0.63 AUC for poor response 0.63 (spherical disproportionality) or 0.61 (heterogeneous texture)
Kim et al. (2017) [50]	- 37 relevant radiomic features selected - Aim: to determine if radiomic features combined to conventional clinical features improved predictive performance in prediction of PFS in EGFR+ adenocarcinoma	48 malignant CT (NSCLC, EGFR mutant)	Prediction of response to TKI - Addition of radiomics to clinical factors improved predictive performance of response to TKI (concordance index: combined model 0.77, clinical model 0.69; *p* < 0.0001)
Lafata et al. (2019) [52]	- 39 extracted features - Aim: to verify the hypothesis that lung texture, in addition to lung density, is partly responsible for correlation between PFT and CT imaging	64 malignant CT (NSCLC)	Prediction of PFTs - Higher DLCO correlated with dense, heterogeneous pulmonary tissue (*p* < 0.002) - Lower FEV1 correlated with homogeneous, low attenuating pulmonary tissue (*p* < 0.03)

CT: Computed Tomography, NSCLC: Non-small cell lung cancer, AUC: Area under the curve, EGFR: epidermal growth factor receptor, KRAS: KRAS gene, CD3: Cluster of differentiation 3, PDL1: Programmed death-ligand 1, SBRT: Stereotactic body radiation therapy, TKI: Tyrosine kinase inhibitor, PFTs: Pulmonary function tests, DLCO: Diffusing capacity for carbon monoxide, FEV1: Forced Expiratory Volume.

**Table 4:** Radiomics and interstitial lung diseases.

Study	Description	Cohort	Performance
Schniering et al. (2019) [74]	- 154 radiomic features extracted - Aim: to evaluate the potential of CT radiomics features for staging experimental ILD and assess transferability to human ILD	66 ILD CT (20 mild ILD and 46 advanced ILD)	Staging of ILD (proof of concept) AUC 0.929
Stefano et al. (2020) [75]	- Extraction of 10 HRCT parameters - Aim: to assess the diagnostic performance of radiomic features in IPF	32 IPF CT	Severity of IPF NL (normally attenuated lung) at -200 HU demonstrated the strongest correlation with disease severity (*p* = 0.009)
Martini et al. (2020) [76]	- 1116 extracted radiomic features - Aim: to retrospectively evaluate if radiomics features are able to detect ILD and distinguish the stages in SSc	66 SSc CT Training cohort 70% Validation cohort 30%	Severity and staging of SSc-ILD - Radiomics features can predict GAP stage with a sensitivity of 84% and a specificity of almost 100%. AUC 0.96. - Correlation of radiomics with GAP stage (but not with the visually defined features of ILD-HRCT)
Ungprasert et al. (2017) [77]	- Extraction of quantitative CT indexes with CALIPER - Aim: To evaluate the correlation between - Quantitative HRCT analysis with CALIPER software and pulmonary function tests (PFTs) in patients with idiopathic inflammatory myopathies (IIM)-associated interstitial lung disease (ILD).	110 ILD CT - 110 baseline CT - 110 1-year follow-up CT	Correlation with PFTs in IIM associated ILD - Baseline: Ground-glass opacities and reticular density had a significant negative correlation with diffusing capacity for carbon monoxide (DLCO), total lung capacity (TLC), and oxygen saturation - 1 year: changes in total interstitial abnormalities had a significant negative correlation with changes in TLC and oxygen saturation
Kim et al. (2015) [78]	- Extraction of quantitative CT indexes (MLA, variance, skewness, kurtosis, median) - Aim: to compare known CT histogram kurtosis and a classifier-based quantitative score to assess baseline severity and change over time in patients with IPF.	57 IPF patients - 57 baseline CT - 57 7-months follow-up CT	Correlation with baseline lung function and prediction of evolution in IPF - All baseline histogram indices (texture features) and QLF and QILD scores were correlated well with baseline FVC and DLCO - When assessing associations with changes in FVC and DLCO over time, only QLF score was statistically significant (*r* = −0.57; *p* < 0.0001 for FVC and *r* = −0.34; *p* = 0.025 for DLCO), whereas kurtosis was not.
De Giacomi et al. (2017) [79]	- Extraction of quantitative CT indexes with CALIPER - Aim: to use quantitative CT analysis to differentiate NSIP versus IPF and assess long-term survival	40 biopsy-confirmed UIP 20 biopsy-confirmed NSIP	Differentiation NSIP vs. IPF - Compared with NSIP, IPF patients experienced greater functional decline (CVF, *p* = 0.02) and radiologic progression (reticulation volume, *p* = 0.048). - Both baseline and short-term changes in quantitative radiologic findings were predictive of mortality.
Lee et al. (2018) [80]	- Aim: to assess quantitative imaging in the evaluation of lymph nodes in pulmonary sarcoidosis and tuberculosis	26 CT from tubrcolosis patients, 21 CT from sarcoidosis patients.	Differentiation between tuberculosis and sarcoidosis LN - Significant differences in the values of the Feret’s diameter, perimeter, area, circularity, mean grey value, SD, median, skewness, and kurtosis between tuberculous and sarcoid LNs (*p* < 0.05)
Best et al. (2008) [81]	- Extraction of quantitative CT indexes (MLA, skewness, kurtosis) and visual scores (fibrosis, GGO, emphysema) - Aim: to retrospectively evaluate quantitative CT indexes as predictors of mortality and describe 12-months changes in CT in IPF patients	167 IPF patients - 167 baseline CT - 167 1-year follow-up CT	Prediction of mortality and progression in IPF - FVC (*p* = 0.006) and fibrosis (*p* = 0.002) were predictors of short-term mortality - Fibrosis index (*p* = 0.03), Mean Lung attenuation (*p* = 0.003), skewness (*p* < 0.001) and kurtosis (*p* < 0.001) predicted disease progression
Maldonado et al. (2014) [82]	- Extraction of quantitative CT indexes and their mean volumetric quantification - Aim: to verify the hypothesis that short-term radiological changes may be predictive of survival by the use of novel software tool CALIPER (computer-aided lung informatics for pathology evaluation and rating)	55 IPF patients	Correlation between CT changes and mortality in IPF - Interval change in quantitative volumetrics (*p* < 0.05) as quantified by CALIPER were predictive of survival after a median follow-up of 2.4 years
Jacob et al. (2017) [83]	- Extraction of quantitative CT indexes to define a quantitative lung fibrosis score (QLF) - Aim: to compare computer algorithm CALIPER to convention CT and PFTs for mortality prediction in IPF	283 IPF CT	Prediction of mortality in IPF - Independent predictors of mortality were CPI “composite physiologic index” (*p* < 0.001) and the following two CALIPER parameters: pulmonary vessel volume (*p* = 0.001) and honeycombing (*p* = 0.002)
Kim et al. (2011) [84]	- Extraction of quantitative CT indexes - Aim: to assess the efficacy of cyclophosphamide in SSc-ILD using texture-based scores (impact on QLF)	83 SSc-ILD CT - 83 baseline CT - 83 1-year follow-up CT	Evaluate the effectiveness of cyclophosphamide in SSc-ILD - Between-treatment - Difference in whole-lung QLF was ~5% (*p* = 0.0190). - Significant associations between changes in QLF and FVC (*r* = −0.33), dyspnea score (*r* = −0.29), and consensus visual score (*p* = 0.0001).

CT: Computed tomography, ILD: Interstitial Lunge disease, HRCT: High resolution computed tomography, AUC: Area under the curve, IPF: Idiopathic Pulmonary Fibrosis, SSc: Systemic sclerosis, CALIPER: Computer-aided lung informatics for pathology evaluation and rating, PFTs: Pulmonary function tests, MLA: Mean lung attenuation, QILD: Quantitative Interstitial Lunge disease, NSIP: Nonspecific interstitial pneumonia, QLF: Quantitative Lung Fibrosis, FVC: Forced vital capacity, CVF: Cobra Venom Factor, SD:standard deviatiosn, LNs: Lymph nodes, GGO: Groudn Glass Opacity and CPI: composite physiologic index.

## Abbreviations

AI	Artificial Intelligence
CT	Computed Tomography
MRI	Magnetic Resonance Imaging
PET	Positron Emission Tomography
DL	Deep Learning
IPF	Idiopathic Pulmonary Fibrosis
CPFE	Combined Pulmonary Fibrosis and Emphysema
QIN	Quantitative Imaging Network
VTD	Volume Doubling Time
SSPNs	Small Solitary Pulmonary Nodules
AUC	Area Under the Curve
HRCT	High-Resolution Computed Tomography
CNN	Convolutional Neural Networks
CAD	Computer-Aided Detection/Diagnosis
NLST	National Lung Screening Trial
EGFR	Epidermal Growth Factor Receptor
NSCLC	Non-Small Cell Lung Carcinoma
GTV	Gross-Tumor Volume
TKI	Tyrosine Kinase Inhibitors
COPD	Chronic Obstructive Pulmonary Disease
HU	Hounsfield Unit
PRM	Parametric Response Mapping
PFTs	Pulmonary Function Tests
LVR	Lung Volume Reduction
ICS	Inhaled Corticosteroids
ILD	Interstitial Lung Diseases
NSIP	Non-Specific Interstitial Pneumonia
CALIPER	Computer-Aided Lung Informatics for Pathology Evaluation and Rating
FVC	Forced Vital Capacity
QLF	Quantitative Lung Fibrosis
PH	Pulmonary Hypertension
PAH	Pulmonary Arterial Hypertension
RHC	Right Heart Catheterization
ROI	Region of Interest
